# A Novel LIBS Sensor for Sample Examinations on a Crime Scene

**DOI:** 10.3390/s24051469

**Published:** 2024-02-24

**Authors:** Violeta Lazic, Fabrizio Andreoli, Salvatore Almaviva, Marco Pistilli, Ivano Menicucci, Christian Ulrich, Frank Schnürer, Roberto Chirico

**Affiliations:** 1 Italian National Agency for New Technologies, Energy and Sustainable Economic Development (ENEA), Laboratory FSN-TECFIS-DIM, Via Enrico Fermi 45, 00044 Frascati, Italy; 2 Italian National Agency for New Technologies, Energy and Sustainable Economic Development (ENEA), Laboratory FSN-FUSEN-TEN, Via Enrico Fermi 45, 00044 Frascati, Italy; 3 Fraunhofer Institute for Chemical Technology ICT, Energetic Materials Department, Joseph-von-Fraunhofer-Str. 7, 76327 Pfinztal, Germany

**Keywords:** LIBS, laser spectroscopy, portable instrument, forensic, crime scene, traces, fingerprints, gunshot residue, sensitive, limit of detection

## Abstract

In this work, we present a compact LIBS sensor developed for characterization of samples on a crime scene following requirements of law enforcement agencies involved in the project. The sensor operates both in a tabletop mode, for aside measurements of swabbed materials or taken fragments, and in handheld mode where the sensor head is pointed directly on targets at the scene. The sensor head is connected via an umbilical to an instrument box that could be battery-powered and contains also a color camera for sample visualization, illumination LEDs, and pointing system for placing the target in focus. Here we describe the sensor’s architecture and functionalities, the optimization of the acquisition parameters, and the results of some LIBS measurements. On nano-plotted traces at silica wafer and in optimized conditions, for most of the elements the detection limits, in term of the absolute element masses, were found to be below 10 picograms. We also show results obtained on some representative materials, like fingerprints, swabbed soil and gunshot residue, varnishes on metal, and coated plastics. The last, solid samples were used to evaluate the depth profiling capabilities of the instrument, where the recognition of all four car paint layers was achieved.

## 1. Introduction

In the last decade, Laser-induced Breakdown Spectroscopy (LIBS) [1] has gained much interest as a tool for in situ or laboratory examination of forensic items [2,3] thanks to its capability to perform rapidly multi-elemental detection in top sample layers, including trace materials on a solid substrate. Different surface analysis techniques used in forensic science, already established or still evolving to be accepted in court, have been recently reviewed [4]. Here, LIBS is considered as one of the emerging techniques because of its high measuring speed, spatial resolution, analyte coverage, as well as the instrument portability to the crime scene and the cost effectiveness. In laboratory conditions, the element detection sensitivity, accuracy, and precision obtained by the LIBS technique are comparable to that of the other spectroscopic instruments, like inductively coupled plasma–optical emission spectroscopy (ICP–OES), laser-ablation inductively coupled plasma to mass spectrometry (LA-ICP-MS), X-Ray fluorescence (XRF), and scanning electron microscopy with energy dispersive X-ray spectroscopy SEM-EDS [5,6,7,8]. LIBS is micro destructive inside the laser spot, typically of diameter in order of 10–100 µm, which is considered sufficiently non-invasive for most of the forensic applications. Its capability to provide chemical information from sample masses below 1 µg makes the LIBS technique suitable for analysis of trace materials, like particles adhering on a solid target or thin ink layer on paper. Furthermore, the spectral detection during layer-by-layer laser ablation at a fixed sample point can provide in-depth element distribution in heterogeneous samples.

The examination of paper material, inks, and toners is very important in any criminal investigation involving documents. A recent pan-European forensic round robin study on document forgery reported comparative results regarding the discrimination of papers, pen signatures, and toners by various analytical tools where only LIBS provided all correct responses in the blind test [5]. In studies of the questioned documents, determining the chronology of deposition of inks on paper is of outmost importance, and this task, difficult also for complex laboratory instruments [9], has been achieved by LIBS [10]. 

Chemical characterization of fingerprints is important because it strongly varies between individuals [11], and it can reveal the presence of forensically important trace materials like explosives, drugs, gunshot residue (GSR), as well as exogenous metals [12]. Different published works have demonstrated feasibility of analysis of fingerprints by LIBS, including their deposition order [13] and material classification from particles initially present on skin and purposely or accidentally transferred to another substrate [6,14,15,16,17]. In case of GSR, the material classification by LIBS is based on detection of Pb, Ba, and Sb in sample, plus other elements commonly associated with modern ammunition (e.g., Al, Ca, Fe, Si, Sn, Zn, Cu, Ti, Sr etc.) [6,17,18,19,20,21]. Differently, recognition of explosives by LIBS where the main elements in the sample are C, H, N, and O, the first two present in organic compounds and the last present in the air surrounding, requires adopting some discrimination strategy based on the line intensity ratios, chemometrics or spectral changes caused by laser induced micro-explosion on energetic materials [14,15,16,22,23]. By using a standoff LIBS system, the analysis of targets, including hazardous materials of forensic interest, can be performed at a safe distance [14,15]. 

In a crime scene investigation, hair is one of the most common traces, and it can vary widely between individuals, making it important evidence in crime scene investigation [24]. The most interesting LIBS approach for forensic examination of hair, requiring a very small amount of sample, regards direct hair probing under a microscope. In this way, it was possible to quantitatively analyze mineral content (Mg, K, Ca, Na, and Al) in concentrations also below 1 ppm [25]. In other works, reporting the chemical composition of hair measured by LIBS, the necessary sample quantity was larger, but the sample preparation remained relatively simple [26,27].

Glass fragments are another type of forensic material, and they are usually chemically characterized by XRF, ICP-MS or LA-ICP-MS techniques. Hower, the discrimination capability of LIBS has been proven to be analogous to the already established techniques [8,28,29]. Combination of LIBS and measurements of refractive index [30] or molecular bonds by Raman [31] helps fast screening of glass micro-fragments and provides additional information for their classification. 

Polymers are encountered as forensic evidence in a variety of materials and regard paints, fibers, tapes, adhesives, and plastics [32]. A large variety of polymers can be classified by LIBS [33,34], including microplastic particles [35]. Paint fragments found on a scene are of particular interest in incidents involving auto vehicles where their examinations [36] can provide information about the make, model, and year of a suspect’s vehicle. Automotive paint typically contains four layers: electrocoat primer on metal, primer surfacer, basecoat, and a clear (top) coat. Among the most interesting tools for chemical analysis of layered materials, there are micro-XRF technique [37] and LIBS [38], where the latter has an advantage of a higher analytical sensitivity, detecting also light elements and having much better in-depth resolution [39].

Instruments to be employed for chemical analysis on a crime scene should provide fast answers to support investigations, also at expense of selectivity—in such cases the results must be confirmed by the measurements repeated in the laboratory. Due to a large variety of materials important in forensic investigations, an ideal LIBS instrument should cover a wide wavelength range and have high spectral resolution in order to identify a large number of elements in the sample, also in the presence of spectral interferences. The sample’s availability on a crime scene is usually in traces, meaning that the LIBS tool, which is micro-destructive, should provide chemical identification by applying a single or a very few laser pulses. The laser pulse energy here should be sufficient to generate an intense LIBS signal, and these basic requirements exclude the use of light handheld LIBS instruments, commercially available and more appropriate for analysis of bulk fragments than trace materials because the extended signal accumulation is necessary [40]. On the other hand, handheld instruments allow for performing measurements on large items (e.g., walls) and without swabbing and transfer of forensic traces that increase the possibility of contamination and compromises the preservation of other forensic traces (e.g., fingerprints). However, direct probing of traces on unknown substrates presents analytical issues due to influence of the substrate on the LIBS signal intensity and substrate ablation simultaneously with a trace sample on it. A recently developed portable LIBS instrument for detection of GSRs [18,19] contains a relatively high-power pulsed laser that, coupled with wide spectral detection range, allowing for a high analytical sensitivity on trace materials. This instrument can be operated both in a handheld mode or mounted on a laboratory scanner, but it might present operational difficulties in the field due to lack of a precise pointing on target. Another, highly performing LIBS instrument that could be transported on a crime scene was developed for detection of swabbed GSR particles [17]. This quite large and heavy mobile device is mounted on a cart that incorporates the computer, instrument itself, and two gas tanks into a single unit that can be transported to the scene by a van. In this case, the transferred forensic particles can be precisely pointed to analyze, thanks to a high magnification camera and a motorized stage. In view of these considerations, we identified a technical gap in the LIBS instrument development for analysis on a crime scene, where the missing tool should provide both handheld and static measurements, not only on GSR but on a wide range of materials, and it should incorporate a camera for sample visualization plus a pointing system for placing targets in the laser focus. 

In this work, we present a novel LIBS prototype, developed to fill some of the existent instrumental gaps and projected to satisfy user requirements identified by law enforcement agencies (LEAs) involved in the Real-time on-site forenSic tracE qualificatioN (RISEN) project. The RISEN system aims at supporting crime scene investigations through the acquisition of an interactive 3D model of a crime scene, which also displays the results of trace analysis obtained by various on-site contactless sensors [41]; among them is another tool developed by ENEA, namely the Crime Light Imaging (CLI) sensor for stand-off photography of latent traces [42]. Here, we also report the results of the LIBS instrument testing aimed to optimize the acquisition parameters, estimate the limits of detection (LODs) for each element, and show the instrument capability to analyze some common trace samples, as well as to identify various sample layers during the depth profiling of paints and coated plastics. 

## 2. LIBS Sensor Development

In the following we describe the LIBS instrument and its functionalities, while the instrument performances in static measurements are reported in the Results section. After optimization of the acquisition parameters, the detection sensitivity and repeatability were evaluated on spots from a 21-element standard plotted on silica wafer in absolute mass between 10 pg and 500 pg per element. Further tests were performed on various traces related to a generic crime scene, like fingerprint, soil, and gunshot residues (GSR). The capability of the LIBS instrument to perform the depth profiling of bulk materials was tested on white car varnishes and coated black plastic.

### 2.1. Input Requirements and Main Hardware Components 

Basing on the inputs from LEAs involved in the RISEN project [41], our design for realization of the LIBS instrument prototype was shaped to comply with the following main requirements:

- The sensor should allow detecting of a wide range of forensic traces and provide in- depth analysis of solid sample fragments. This requirement was translated into a choice of a relatively high energy pulse laser and the detection system covering a wide spectral range with high spectral resolution.
- The sensor should have a possibility to be operated both in a static (tabletop) and handheld mode. For this reason, the sensor was projected to have a detachable, relatively light head connected via a 2 m long umbilical to the instrument box.
- The sensor should work both in indoor and outdoor environments. The influence of the environmental conditions is minimized through a short working distance (<5 cm) and thermal stabilization of the spectrometers.
- Due to the expected small availability of trace materials on a crime scene and micro-destructive nature of LIBS, the instrument pointing should be precise. This capability was achieved by providing high magnification live images of samples and by two pointing lasers.
- The instrument functioning should have a relatively low power consumption, allowing for at least one hour of battery operation. For this reason, the most power-consuming component, namely the high-power laser, was chosen to be diode-pumped due to its high conversion efficiency from the plug to optical power.
- The instrument deployment and use are expected to be relatively fast and simple, with a user friendly Graphical User Interface (GUI).
- The results of the measurements should be almost immediate, and together with the case identification data, measuring parameters, instrument status, and annotations from operator, they should be transmitted to the central RISEN 3DA-CSI system that collects the data from the various sensors employed on a crime scene.

The main functional hardware components of the herein presented LIBS sensors are

1. Laser system;
2. Detection system;
3. Pointing and viewing system;
4. Control system;
5. Structure for table-top measurements (optional).

**Figure 1:**
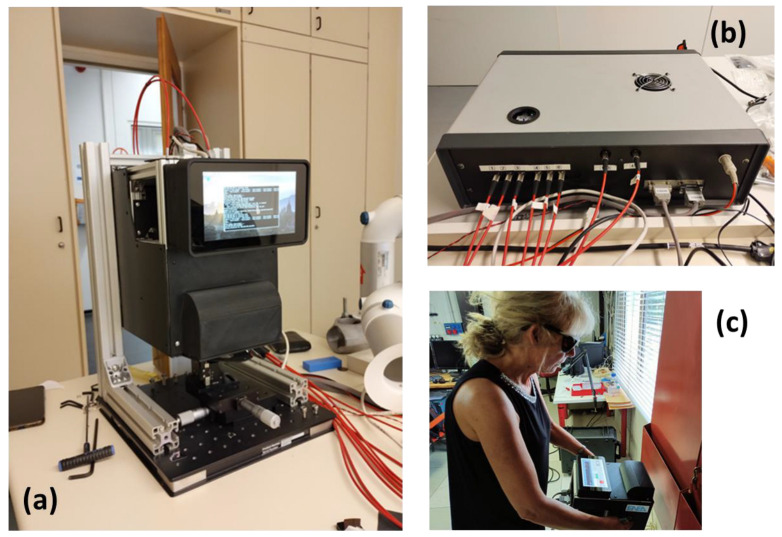
(**a**) LIBS sensor head mounted on a structure for static measurements; (**b**) the instrument box; (**c**) sensor head operated in a handheld mode.

The aspect of the LIBS sensor used in a static mode during the first RISEN trial (October 2023, Pfintzal, Germany) is shown in Figure 1a,b. The sensor head is fixed on a static structure by four screws. Operation of the sensor in a handheld mode is depicted in Figure 1c where, after establishing the communication between various instrument components, the user can visualize the GUI on the LCD screen as an alternative to a full-screen sample image. To facilitate the instrument deployment in the field by a single operator, all connecting cables between the sensor head, instrument box, and control laptop (if used) are properly labeled. The instrument box is powered by 24 VDC or 220 VAC. 

The dimensions and weights of the sensor’s modules are given in Table 1, which are reduced compared to the most similar portable LIBS instrument [16], although having also the built-in camera with target pointing and higher performances in terms of laser power density, spectral range, and resolution.

The details about each of the main components are given in the following sections. 

#### 2.1.1. Laser System

The laser source for generating the LIBS signal is a diode pumped Nd:YAG laser by BrightAerospace, model BAS_CDL-1064-50mJ-DB. The laser emits pulses of 6.5 ns duration at the wavelength of 1064 nm, with the repetition rate between 1 Hz and 5 Hz; the maximum pulse energy is of 50 mJ. The laser power supply, operating at 24 VDC, was placed inside the instrument box and connected to the laser resonator inside the sensor head via a 2 m long umbilical. To minimize the time jitter, we exploited the available external trigger inputs to control both the start of the laser pumping and the Q-Switch opening, delayed for 200 µs from the first trigger.

Due to lateral diode pumping of the active laser material, the output laser beam was rectangular, with dimensions of 5 mm × 7 mm, corresponding to the beam divergence of 0.5 mrad and 0.7 mrad. The laser beam was bent by laser mirrors and expanded and focused by a lens with focal length of 100 mm. The choice of such a relatively long focusing distance was guided by a need to have sufficient depth of focus in handheld operating mode; this choice also keeps low optical aberrations of the viewing system, without need for a specially designed objective for the color camera. 

The sensor head components were pre-aligned for placing the target surface at a distance of 12.6 mm from the exit and above the laser focus, in a way to generate a spot of equivalent diameter of 0.30 mm. In such conditions, considering the measured optical losses, the maximum energy density on the target was of about 57 J/cm^2^, equivalent to instantaneous power density of 8.7 GW/cm^2^. 

#### 2.1.2. Detection System 

The optical collection of the plasma emission consists of two identical systems based on off-axis parabolic mirrors with UV enhanced aluminum coating. Each of these two systems focuses the signal on a fiber bundle containing four quartz fibers with diameter of 600 µm. Three of these fibers are used to transmit the signal to the spectrometer channels, while the fourth one is exploited to launch the light from a beam pointer onto the sample. The fiber bundles are 2 m long, and the single fibers are labeled to be connected always in the same way because of small mutual shift between pointing of single fibers on target; the optical alignment was performed in a way that the collected LIBS signal on various spectrometer channels has no significant jumps in the detected plasma continuum emission. 

The plasma detection is based on six compact spectrometers (Avantes, model AvaMINI 2048), customized to cover the range from 180 nm to 875 nm, with higher spectral resolution in the UV region than at longer wavelengths. The spectrometers have an internal coated lens that allow the signal collection from 600 µm large fibers, and they were purchased with 10 µm large entrance slits. The characteristics of the single spectrometers are summarized in Table 2, where the spectral resolution was determined by a calibration HgAr lamp, considering full width half maximum (FWHM) of the detected lines. Each spectrometer was initially calibrated, and the acquired spectral data from different channels are automatically unified by a home-built software, taking only the spectral ranges (used range) selected in a way that the cut wavelengths do not coincide with line peaks in multi-elemental samples. The data taken to determine the spectrometer resolutions were obtained by the original producer’s software (Avasoft 8.14.0.0), where for determination of the FWHM for the first spectrometer the Hg I line at 253.65 nm was considered.

The detectors have a minimum integration time of 30 µs, and here they are externally triggered and synchronized. The spectrometers were tight together inside the instrument box, occupying volume of 95 × 68 × 120 mm^3^ with total weight of 1.05 kg. On the top of the spectrometer array, we placed a Peltier’s plate stabilized at 22 ± 0.5 °C, thus avoiding the necessity to repeat wavelength calibrations during the excursions of ambient temperature. 

#### 2.1.3. Pointing and Viewing System 

**Figure 2:**
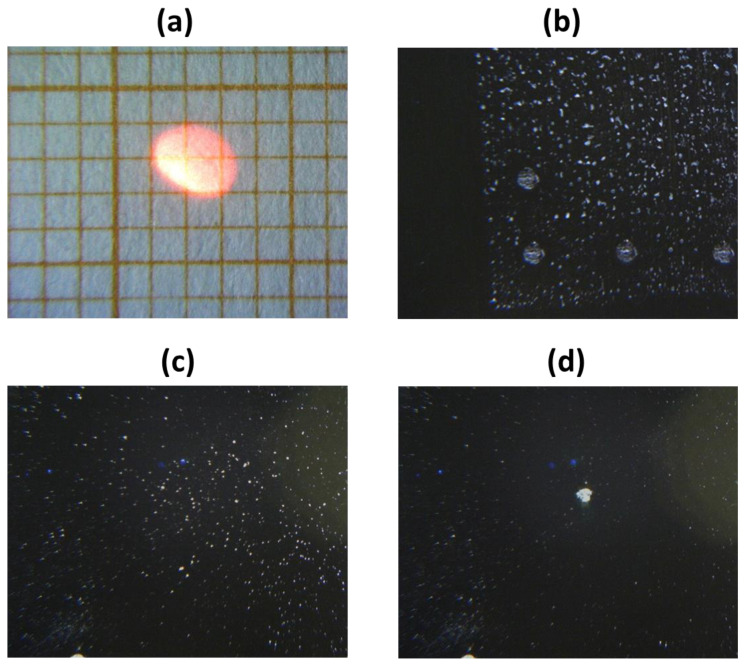
Photos taken by the instrument camera on (**a**) millimetric paper with one pointer turned ON; (**b**) PETN printed on Al substrate with 100 µg/cm^2^ where the previously induced laser craters by five pulses are present—the photo was captured without white LED illumination. (**c**) PETN particles transferred on Si wafer before and (**d**) after the LIBS measurement—the photos were taken with white LED illumination.

For visualization of a target, a compact color camera with objective is mounted coaxially to the exiting laser beam. The camera sensor has 1280 × 1024 pixels and for the optimally aligned LIBS instrument, the captured sample area is of 19.0 × 15.2 mm^2^ (Figure 2a), corresponding to the theoretical spatial resolution of 1.5 µm, which is sufficient to observe micrometric size particles (Figure 2b,c). On the Si wafer, the laser-induced craters are of smaller diameter (Figure 2d) than on the Al target because of higher melting temperature of the dielectric material and reduced heat effected area by the laser pulse. 

When operating the LIBS sensor via the instrument’s GUI (see Section 2.2), the last frame before the laser shooting is automatically stored while the additional photos, for example after the measurement, could be taken as well. The sample can be illuminated by turning on an array of white LEDs placed inside the instrument head. 

To have an accurate sample pointing, on the images viewed by the LCD a circle indicating the laser spot was added via software, not appearing in the automatically saved images. 

The pointing system is exploited to set the correct distance between the sensor head and sample, and it is particularly important for handheld instrument operation. The two pointing diodes, placed inside the instrument box, have peak emission at 635 nm with CW power of 4.5 mW. The diode emission is optically coupled to a fourth fiber from the bundle used to transmit the plasma emission to the spectrometers. The red spots on a sample, generated by the pointers, are crossed if the sensor-to-target distance is optimal. 

#### 2.1.4. Control System

**Figure 3:**
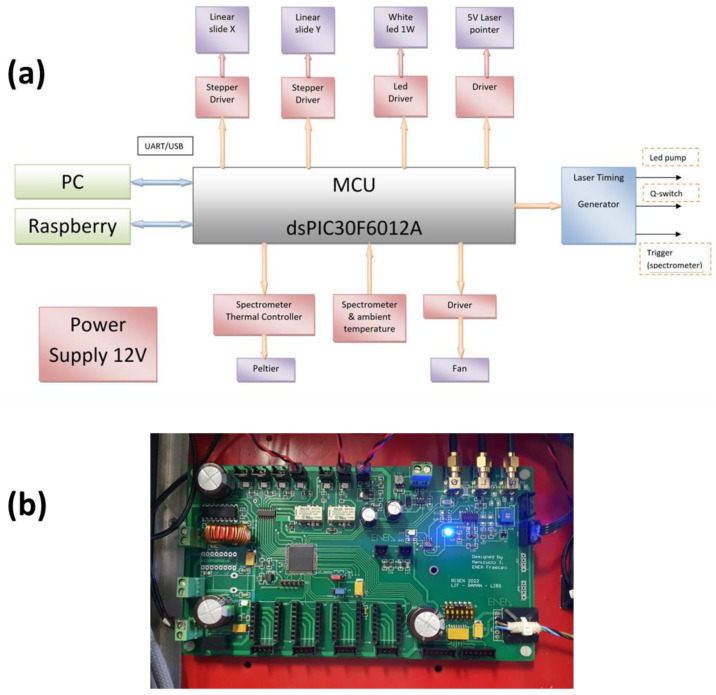
Control system: (**a**) architecture and (**b**) photo of the developed electronic board.

Some of the LIBS sensor’s components are driven by custom electronics developed in ENEA’s laboratory, and the corresponding architecture is shown in Figure 3. The electronic card is based on a dsPIC30F6012A controller, and it is powered by 12 VDC. The card provides the controls of white LED illumination, two laser pointers, temperature measurement inside the instrument box, and activation of the corresponding cooling fan, plus functioning of the Peltier cell mounted on the spectrometers. The same electronic board supplies the triggers for the laser diode pumping and Q-Switch aperture, as well as for the spectrometers. In this way, the time jitter of the laser pulse was stabilized to less than 35 ns, while pre-triggering of the spectrometers compensated the electronic delays inherent to the detecting devices, thus allowing for the effective signal acquisitions already starting from the laser pulse or with the realistic delay from the same. The camera images are locally acquired by a small single-board computer and sent via Wi-Fi or Ethernet to the laptop or the central RISEN data collector.

### 2.2. Software and Graphical User Interface (GUI)

**Figure 4:**
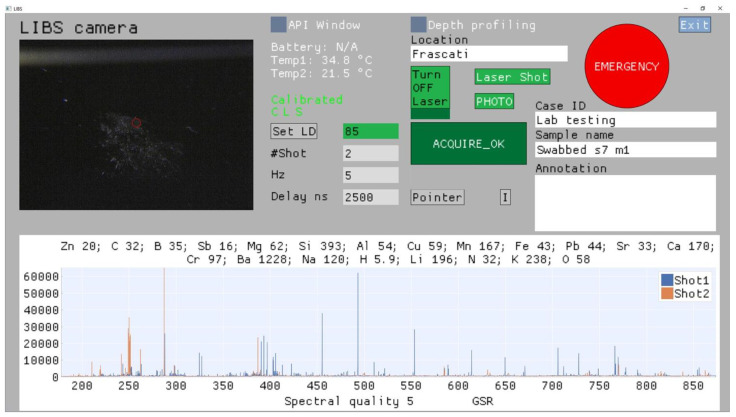
GUI—as it appeared after measurement on a swabbed GSR and before launching a new acquisition on a new sample of the same type, captured by the online camera.

The software design features an intuitive GUI (Figure 4) that provides the instrument operation both via computer desktop and LCD on the instrument head. The GUI also shows the instrument status and displays the results of the measurement. The other menus are also available to be open, like the API window for sending the significant data to the central LEA’s system and the depth profiling window for visualization of the results of multi-pulse measurements at one sample spot. 

After launching the instrument’s software, the connection with the devices is automatically checked, and if correct, the live camera image appears and letters C (electronic Card), L (Laser), and S (spectrometers) turn from red to green color. The laser is automatically turned on, and the pumping Laser Diode (LD) current is set to be 0. If the instrument is connected to battery, its voltage (nominal 24 V) is displayed. The monitored temperature of the raspberry (Temp1) and spectrometers case (Temp2) are also shown. 

The target to analyze can be further illuminated by white LEDs, by pressing the button I. When pointing the target in handheld mode or if the sample height is changed in tabletop mode, the pointers P can be activated—they overlap at the sensor’s focal plane. The image of the sample, with superimposed red circle corresponding to the laser spot, can be expanded across the whole window. 

The measuring parameters to be set are the following: LD current (0–100), number of laser shots per measurement, the laser repetition rate (1–5 Hz), and the effective acquisition delay from the laser pulse (≥0 ns). The operator can also set the case identification (ID), supplied by the central system (by LEAs), then location, sample name, and annotations. All the mentioned information, once the measurement is performed by pressing the button ACQUIRE, are locally stored together with the sensor status, the last camera’s frame before the laser shooting, and results of the measurement. The large EMERGENCY button stops the laser emission (sets the LD current to 0) at any moment, also during the data acquisition.

The measurement results, automatically stored, include the full spectrum after each applied laser shot, spectral quality (0–5) retrieved from the intensity of the strongest detected line peak, list of the detected elements with the calculated Signal-to-Noise Ratio (SNR) for the default analytical lines (see Section 3), and eventual sample identification, presently implemented for the following classes of materials: explosive (including organic precursors), potassium-based compound, and gunshot residue. The corresponding identification algorithm is beyond the scope of the present work. For the default settings, the spectra obtained by the first two laser pulses are displayed, and they can be zoomed, while the detected elements with the corresponding SNRs are shown for the spectrum acquired by the first laser pulse.

After the LIBS signal acquisition, the operator can make additional photos of the sample by pressing the button PHOTO, for example, to see the laser-generated crater and eventual surface removal of swabbed particles. These photos, together with the data from above, are stored in the same directory, whose name contains the data-timestamp plus the given sample name. Inside the directory, each file name contains also the corresponding data-time stamp, important for conservation of forensic evidence. 

## 3. Results of Preliminary Testing of the LIBS Sensor

The first tests of the sensor were performed in a static mode (tabletop) on two types of representative samples, namely—trace materials on wafer substrate, ablated already by one laser pulse, and solid fragments of metals and plastic with surface coating where the depth profiling by LIBS was applied. The spectra taken on the clean wafer substrate are used as a reference for fast checking of the correct instrument functioning. After the optimization of the experimental parameters, the sensor’s detection sensitivity for single elements was estimated from 21-element standard (21ST) plotted with known mass (in picograms) on the wafer surface. Among numerous samples tested by the LIBS sensor, here we report the results of the tests on materials listed in Table 3.

### 3.1. Samples for Testing

The silica wafer used for check of the correct instrument functioning and as a substrate for swabbed particles was 0.55 mm thick and covered by a SiO_2_ layer of 285 nm. The original wafer has diameter of 4 inches, and it was cut in smaller rectangular pieces by a diamond cutter. To avoid the surface contamination, the operator used the proper gloves while eventual residual particles from the cutting were blown by clean dry air. 

**Figure 5:**
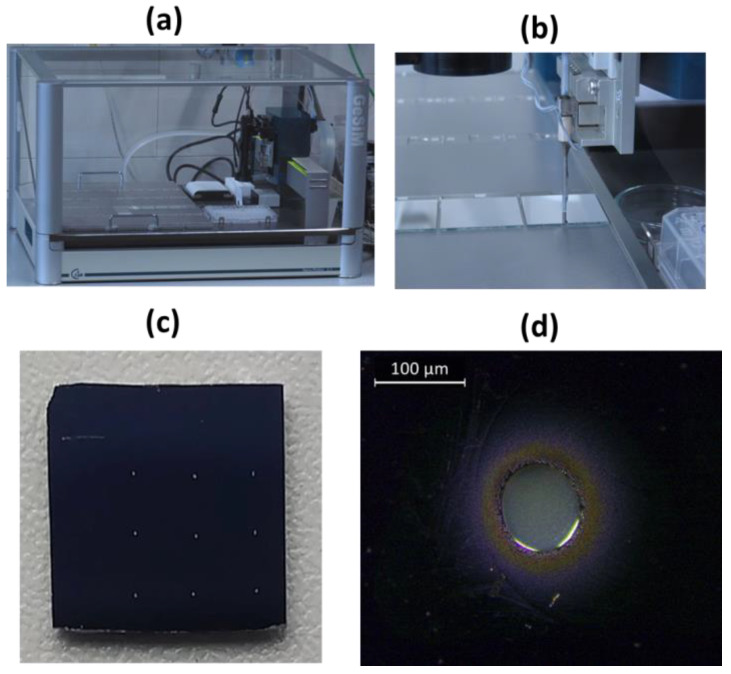
(**a**) Nanoplotter NP 2.1 and (**b**) piezo pipette delivering the droplets on flat substrate. (**c**) Photo of the wafer printed by 500 pg spots. (**d**) Photo of a 500 pg spot taken under optical microscope.

The reference water solution for calibration (21ST) contains 100 mg/L each of the following elements: As, Be, Ca, Cd, Co, Cr, Cu, Fe, Li, Mg, Mn, Mo, Ni, Pb, Sb, Se, Sr, Ti, Tl, V, and Zn, in HNO_3_ 2%/tr. HF, tartaric acid. This standard was further diluted in milli-Q water to prepare solutions with concentrations between 1 mg/L and 10 mg/L of each element. These solutions were used for delivering the spots on wafer by drop-on-demand printing, here performed by a Nanoplotter NP 2.1 from GeSIM (Figure 5a). The printer is equipped with a piezo pipette of the “Nanotip”-type, delivering droplet sizes of 200–400 pL, whose volume is then measured by a stroboscopic view and flow sensor. Electric impulses (50–120 V, frequency 50–1000 Hz) deliver single droplets from the orifice at the tip of the piezo-pipette (Figure 5b). An automatic X-Y-Z stage allows for depositing one or multiple droplets of a printing solution on defined locations of a flat substrate. After evaporation of the solvent, solid deposits remain on the surface. For studies of the LOD s of the LIBS sensor, spots with element loads of 1, 5, 10, 100, and 500 pg were prepared by applying 3–32 droplets on silica wafer, in a grid of 3 × 3 spots distanced 3 mm from each other (Figure 5c). The diameter of the largest printed spot, corresponding to 500 pg load (Figure 5d), was smaller than the laser spot diameter, the necessary condition to probe the whole deposited sample mass by a single laser pulse. Unfortunately, it was not possible to locate the printed sample points for masses below 10 pg because they were too thin to be observed under the sensor’s camera. Consequently, the tests were performed only on samples containing 500 pg (sample 2), 100 pg (sample 3), and 10 pg (sample 4) per element.

Groomed fingerprints containing sebaceous secretions (sample 5) were collected in the following way: the donor’s hands were washed with soap, and fingertips were rubbed across the forehead to saturate the fingertips with sebaceous secretions. The fingertips from both hands were rubbed across one another to homogenize distribution of the groomed secretions among all 10 fingertips. The transfer on the silica wafer was produced by an in-house developed fingerprint sampler containing a lever-mechanism that assured a fixed amount of force (i.e., weight applied at the end of the lever). Using the handle of the lever, the substrate could be easily applied or removed from the finger for a selected duration (i.e., time of transfer). Here, the time of transfer was set to 5 s, while the weight of the lever was 1 kg.

Particles of soil (sample 6) were delivered on the wafer surface by a manual blower with a brush on the tip, from a distance of a few centimeters. The particles were randomly distributed on the surface, with some agglomerations at certain points (see Section 3.3). The laser pulse(s) blow the particles around the spot, so for the repeated measurements it was necessary to distance the points ≥3 mm or to add new particles. The soil sample here examined was NIST2710, having an elevated concentration of metals.

The examined GSR (sample 7) was swabbed directly from a gun and smeared over the wafer surface. The sample contained also particles from the cartridge. The smeared material was sticky and not significantly removed by the laser-induced shockwaves, so it was possible to perform samplings at relatively close points, here distanced 1 mm from the previous ones. 

The first white varnish (sample 8) came on a flat metallic substrate having an area of 10 × 10 cm^2^. The varnish thickness was of about 104 µm, as measured by profilometer Profilm 3D^®^. The other varnish (sample 9), with area of about 5 × 10 cm^2^, had a very similar color to the previous sample, but the measured coating thickness was smaller, of about 34 µm. These two samples were directly probed at both sides, i.e., on varnish and on bare metallic substrate. 

The black ABS with fire resistant (FR) coating (sample 10), as common for the electronic components, came as a piece with area of about 2 × 1 cm^2^. The top side of the sample had a dark grey lucid coating, while the back side, of similar color, had rougher and opaque finishing. This sample was also examined by LIBS at both sides.

### 3.2. Optimization of the LIBS Detection 

The instrument is normally used with the laser pumping below 100%, and all the measurements reported in this work were performed for the LD current set at 85%, corresponding to the laser energy of 31 ± 1 mJ on target. The laser was operated at the repetition rate of 3 Hz, where for the trace characterization we delivered two sequential laser pulses. The signal acquisition gate was set to the minimum, i.e., to 30 µs.

The optimization of the acquisition delay was conducted for trace materials on the wafer, and for this purpose we used printed spots of ST21 where the single-element masses were of 500 pg. The analytical lines considered for each element, here as net peak intensities, are listed in Table 4, reporting also the upper level’s energy E_k_ (eV) of the transition, as well as some relevant data obtained from the tests. Note that some of the selected lines are resonant and might suffer from self-absorption—this should be considered when building the calibration curves, which are beyond the scope of the present paper. 

**Figure 6:**
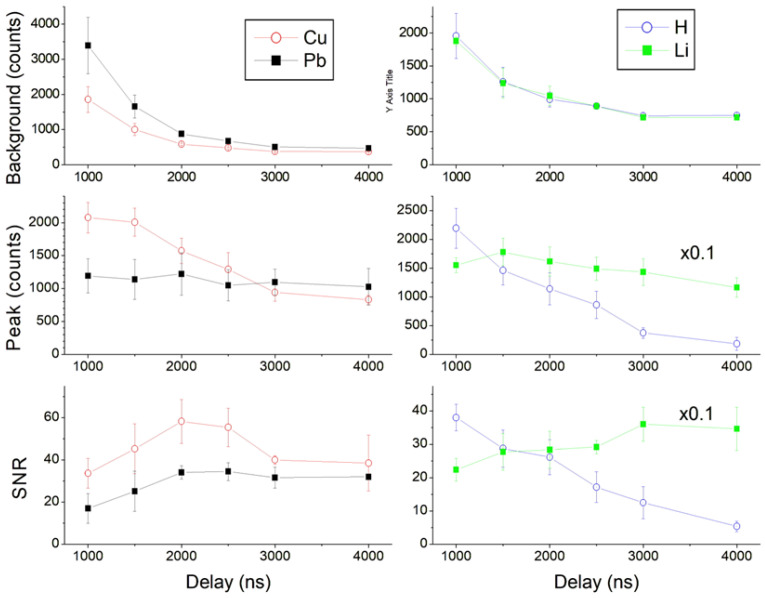
Average spectral values as a function of the acquisition delay for Cu I, Pb I, H I, and Li I lines: first raw—nearby continuum intensity; second raw—net peak intensity; third raw—signal-to- noise ratio (SNR) of the line.

The plasma continuum emission, well visible for short acquisition delays (<2000 ns), had a wide peak at about 450 nm, affecting mostly the spectral lines in the range 380–520 nm. In Figure 6-left, we show behavior with the acquisition delay of the Cu I (324.75 nm, E_k_ 3.817 eV) and Pb I line (405.78 nm, E_k_ 4.375 eV), regarding the nearby continuum plasma intensity, net peak intensity, and signal-to-noise ratio (SNR) of the peak. The continuum emission rapidly decayed with the acquisition delay, and it was less intense in the UV than in the visible spectral region, thus not covering the Cu I peak during the early plasma. The Cu I peak intensity was maximum for the smallest delay here considered (1000 ns). Differently, the Pb I peak, although with a relatively similar E_k_ to that of the Cu I line, did not fully emerge from the plasma continuum component at short delays. The SNR of the Pb I peak increased from delay of 1000 ns to that of 2000 ns, reaching an almost stable value, while the SNR of the Cu I line had the maximum SNR at 2000 ns. The other comparative behaviors, shown in Figure 6-right, regard the H I (656.28 nm, E_k_ 12.088 eV) and Li I line (670.78 nm, E_k_ 1.848 eV), which have very different excitation energies but are spectrally close, although registered at different spectrometer channels (see Table 1). In this case, the plasma continuum intensity had the identical decay (inside the measuring error) with the acquisition delay, but the H I peak dropped rapidly due to the plasma cooling and depopulation of high energy excitation states. This line had the maximum peak intensity and SNR for the shortest tested acquisition delay (1000 ns). On the other hand, the peak intensity of the Li I line, coming from a low excitation level, was almost constant in the measured delay range, while the SNR was maximum between 3000 ns and 4000 ns, where the contribution of noise from the plasma continuum is almost extinguished. 

From the above discussed results, it is evident that both the wavelength and the upper energy level of transition determine the optimal acquisition delay for each analytical line, initially selected to be free of spectral interferences in multi-elemental samples. For example: the well-isolated Zn I (213.86 nm, E_k_ 5.796 eV) had the maximum SNR at delay of 1500 ns, which is reduced by about 30% at 2500 ns; for the Sb I peak (259.80 nm, E_k_ 5.826 eV), important for detection of GSR and close to interfering Fe II lines, the SNR had almost stable values up to delay of 2500 ns, after which it started to decay. The elements present in air (H, N, O) and in high explosives, thus important for their identification, have emission lines from high excitation levels and so they decay rapidly. Consequently, to detect them with a sufficient SNR, the acquisition delay should not exceed 3000 ns.

Following all considerations in this section, as the optimal acquisition delay for detecting of a wide range of elements in a trace sample, we adopted the value of 2500 ns. For the detection of explosives by LIBS, not discussed in the present work, the optimal acquisition delay should be shorter, between 1200 ns and 1500 ns, to obtain a good signal from the atoms that are the main sample constituents (C, H, N, and O).

### 3.3. Detection of Trace Materials by the LIBS 

For the optimal acquisition delay of 2500 ns, on the 21ST sample printed as 500 pg per element, all the certified constituents, except As, Se, and Tl, were detected. In addition to them, traces of Al, C, K, and Na, present in the sample as impurities, were always observed in the spectra plus emission lines of Si, coming from the wafer simultaneously ablated with the trace sample. The same elements as above were still detected in the 21ST sample printed as 100 pg per element, while for the same sample deposited in a mass of 10 pg per element, the lines from Zn, Pb, and Sb were below the detection threshold. Here, the obtained detection sensitivity in single-pulse LIBS measurements could be considered excellent, also in comparison with the previously published results where, for example, LODs were of 0.1–18 ng for each of the target elements after probing spiked materials on Teflon by averaging over 128 laser pulses [6].

**Figure 7:**
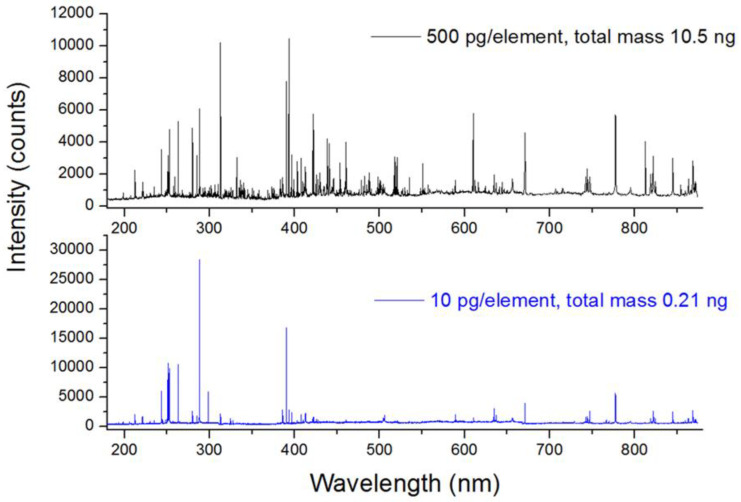
Single-shot spectrum from 21st sample plotted in mass 500 pg and 10 pg per element, equivalent to the total trace mass of 10.5 ng and 0.21 ng, respectively.

The comparative single-shot spectra of samples with 500 pg and 10 pg per element are shown in Figure 7, while the SNR of the detected lines for the three-element masses of 21ST are reported in Table 4. Note that some element peaks had the highest SNR for the intermediate sample mass (100 pg), and this can be explained by a more intense and hotter plasma mediated by the wafer support [43]; consequently, the maximum detection sensitivity on traces requires an optimal, partial substrate coverage. This fact implies that in quantitative LIBS analysis of trace materials it is necessary to take into account the plasma parameters, as suggested in [43,44]. 

The measurement repeatability in terms of Relative Standard Deviation (RSD) of the measured net peaks was calculated from five repeated measurements on the 21ST sample with load 100 pg per element. For most of the analytical lines, the RSD regarding the certified elements in sample was below 0.1 (Table 4, last column). The highest RSD (0.26) was found for the Sb I line that had low SNR, and it was strongly affected by nearby Fe II lines; in samples containing much higher mass proportion Sb:Fe than here (1:1), like in GSR traces, the RSD of this line is expected to be reduced significantly. Due to a high spectral resolution of the LIBS detection system used, the peak fitting did not bring significant improvements in the measurement repeatability.

**Figure 8:**
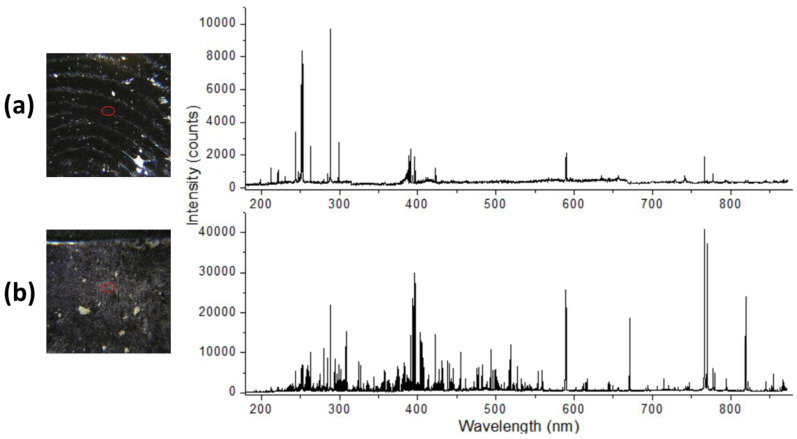
Photo of sample before the LIBS measurement and single-shot spectrum from: (**a**) sebaceous fingerprint and (**b**) particles of soil NIST2710, left on the wafer. The laser spot position is indicated by red circle.

The fingerprint on wafer (sample 5) was well visible under the instrument’s camera, and the laser spot was larger than the fingerprint ridges (Figure 8a). From single-pulse measurements, repeated at 10 points distanced at least 1 mm to each other, it was possible to detect atomic emissions from C, Ca, Na, Mg, and Sr in sample, plus weak lines from Al and Li. The CN UV band was also present in the spectra. 

On the soil particles (sample 6), the spectral intensities depended on the local surface coverage, but generally, the detected emission was extremely rich in lines (Figure 8b); in the reported spectrum, the following elements in the sample were detected: Al, B, Ba, C, Ca, Cr, Cu, Fe, Li, K, Mg, Mn, Na, Pb, Sr, Ti, V, and Zn.

**Figure 9:**
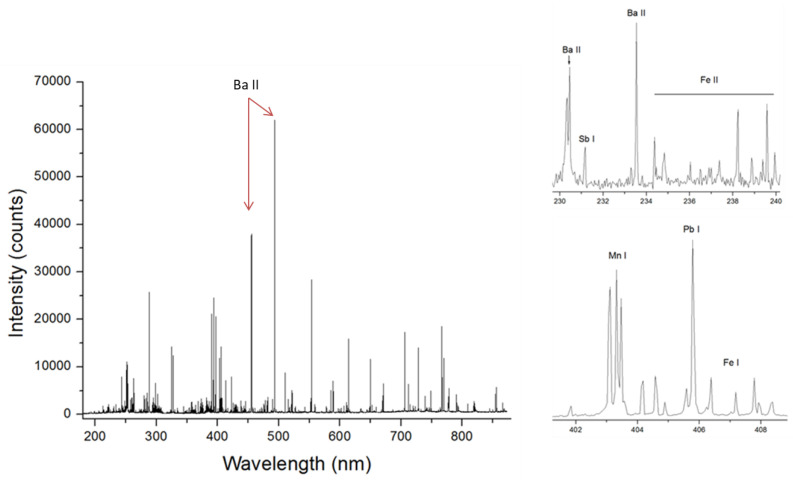
Full single-pulse spectrum of the GSR (sample 7) and some characteristic spectral details.

On the swabbed GSR residue (sample 7), the single-shot LIBS spectra were very intense (Figure 9), showing the presence of the following elements: Al, Ba, C, Ca, Cr, Cu, Fe, K, Mg, Mn, Na, P, Sr, and Zn; and a weak signal from Ni in some sampling points. The most intense lines in the spectra belonged to the Ba II transitions. The Sb was visible from its various atomic lines. 

### 3.4. Depth Profiling by the LIBS

The two varnishes on metal had very similar aspects. The measurements were performed both on the paint, by 100 laser pulses, and at the sample’s back (uncovered) by five subsequent pulses.

**Figure 10:**
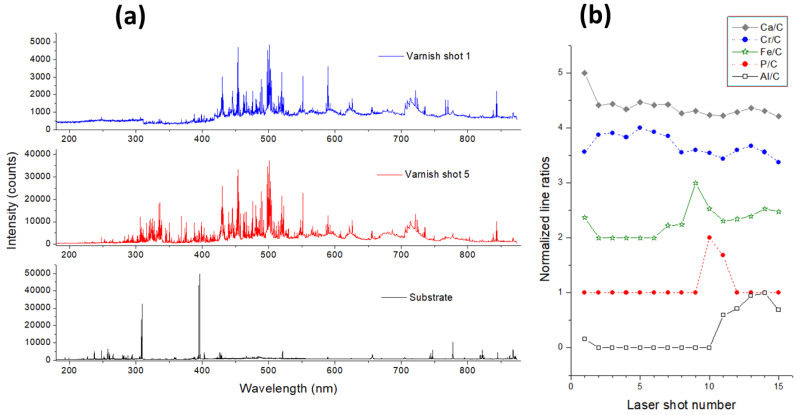
(**a**) Single-pulse LIBS spectra obtained on varnish topcoat (shot 1), basecoat (shot 5), and substrate of sample 8. (**b**) Normalized line intensity ratios Ca/C, Cr/C, Fe/C, P/C, and Al/C as a function of the laser shot number during the depth profiling of varnish.

The metallic substrate of varnish sample 8 was based on Al alloy containing Mn, Cr, Si, and C where, after three cleaning pulses, weak emissions from Cu, Fe, Mg, Na, and K were still present; thus, these elements could be considered as impurities in the alloy. The LIBS spectra taken on the front, painted sample side were dominated by Ti I and Ti II lines, and with well-detectable emission from C I in the UV region. The intense Al I emission occurred starting from the 11th laser pulse, indicating that the metallic substrate was reached through laser ablation. The ablation rate of metal being lower than that for the varnish, the successive pulses produced the enlargement of the laser-induced crater, found to have the mean diameter of about 550 µm and depth of 104 µm at the end of the measurement. Due to the crater enlargement, the emission from varnish constituents persisted where their line peaks dropped down to stable values after about 40 laser pulses. The LIBS spectrum on the topcoat varnish layer was weak compared to the spectra taken by the successive pulses (see Figure 10a). Through analysis of the characteristic element peaks as a function of the laser shot number (Figure 10b), it was possible to observe four varnish layers. The topcoat, in addition to C and Ti, the latter element possibly attributed to a simultaneous partial ablation of the underneath paint, contained mainly Ca, Na, and K plus a very weak emission from Al. The successive paint layer (basecoat), probed by the second to seventh pulses, contained also Ba, observed from a weak peak at 493.41 nm, then Cr, Cu, Mg, and Si. From the seventh laser pulse, emission lines from Fe appeared, reaching the maximum intensity for the ninth shot, thus indicating a presence of an iron-based coat (surfacer). For the 10th and 11th pulses, a presence of phosphor, attributed to a thin conversion layer (primer), was evident from the P I line at 253.56 nm, free of the spectral interferences in this sample. From the 11th laser pulse, the monitored Al I peak appeared, indicating that the interface between the primer and metallic substrate was reached. The estimated average ablation rate across all paint layers was of about 10 µm/pulse.

The probed substrate of sample 9, according to the LIBS analysis, was of a Ti-Zn alloy containing Mn, plus impurities of Fe, Ca, Na, and K. The varnish was also based on Ti and C, but it also contained a significant amount of Ba, visible through various ionic lines from UV to the red spectral range. The presence of Ca and K was much more pronounced than in the previous varnish sample and accompanied by relatively intense lines from Sr II; the elements detected in traces were Mg, Si, Fe, and Na. The Zn-containing substrate appeared from the fifth pulse together with the P emission, but the transition between the various paint layers was less sharp than on sample 8, probably due to surface curvature of the metallic sheet. The estimated average ablation rate across the paint was of about 8 µm/pulse.

**Figure 11:**
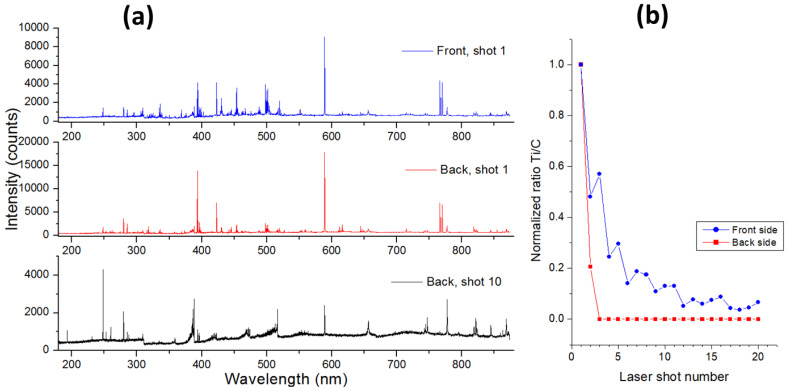
(**a**) Single-pulse LIBS spectra obtained by the first laser shot on the front and back side of sample 10 (ABS-FR), and the spectrum from bulk materials taken for the 10th pulse at sample’s back. (**b**) Normalized line intensity ratio Ti/C measured on both sample sides.

The black ABS sample was examined by LIBS at both sides of the plastic sheet, and the corresponding spectra are shown in Figure 11a in comparison with the same obtained on the bulk plastic material. On the back side, the bulk material was reached after two laser pulses, where the emission lines from Ti disappeared and the spectra taken by the successive pulses mainly contained C I lines, CN and C_2_ bands, plus the emission from Al, Ca, Na, K, Mg, Si, and Sb. Except for Sb, the line peaks from these elements normalized on the C peak, decaying in depth, indicating the crater enlargement that involved the thin surface coating. Differently, Sb, here observed through various atomic transitions, was a part of the bulk material as its line intensity normalized on the C I peak and fluctuated around a stable value across the sample depth. On the front side of the same plastic sample, for the first few pulses the emission lines from the FR coating (Ti, Al, Ca, Mg, K, and Si) were more intense than on the back side, and the traces of Fe were also observed. The decay of Ti/C peak ratio in depth was gradual (Figure 11b), reaching a stable value approximately after 12 laser pulses, thus indicating a much thicker FR coating than on the back sample side. 

## 4. Conclusions

In this work, we presented the first prototype of a compact LIBS sensor, developed at ENEA for analyzing samples on a crime scene, both directly—in a handheld operating mode, and out of the scene—on swabbed traces transferred to silica wafer or of solid fragments. Operating the instrument does not require an expertise in LIBS, thanks to the user-friendly GUI that also monitors the instrument status, shows the live magnified image of the sample, helps the correct focusing on target, and provides automatic data elaboration and storing. The software also offers menus for further visualization of results, like those of depth profiling, and for the data transfer to the central system that collects the results from various sensors employed in the RISEN project. 

The LIBS signal is acquired over a wide spectral range, allowing for multi-elemental detection on various samples of forensic interest, also by applying a single laser pulse. Preliminary testing of the sensor, after optimization of the experimental parameters, showed an excellent detection sensitivity below 10 picograms of absolute mass for most of the elements in traces deposited on silica wafer. The other representative trace samples were also examined, confirming the sensor’s capability to retrieve a large amount of information about their chemical composition. The depth profiling of car paint revealed all four sample layers, while on a plastic sample with fire resistant coating, it was possible to observe differences in the coating thickness at two examined sample sides. 

Future testing of the LIBS sensor concerns extensive measurements on various samples and substrates that could be found at a crime scene, both in the laboratory and during scheduled final trials of the RISEN project. Regarding the device’s development, the upgrade of the present prototype includes the size reduction of the instrument box to fit into a backpack together with batteries, introduction of motorized slits for sample positioning when operating the instrument in tabletop mode, and increase in the spatial resolution of the viewing camera. The software will be further upgraded according to the suggestions received from LEAs, and the automatic data elaboration will be improved and extended to identify more elements and sample classes than now; an estimation of relative element concentrations in samples will also be introduced in the near future. 

Although the LIBS instrument here was realized for investigations on a crime scene, where it is necessary to track and submit the data to the central system, its possible applications include various types of materials and in situ analysis of objects from cultural heritage, environmental monitoring, and elsewhere. The authors are aware that portable LIBS instruments, including the herein presented prototype, should pass extensive scientific review and method consolidation before being accepted in routine forensic workflows.

## Figures and Tables

**Table 1:** Dimensions and weight of the LIBS sensor modules without batteries.

Module	Dimensions (cm)	Weight (kg)
Sensor head	20 × 28 × 27	3.3
Instrument box	51.5 × 16.5 × 12	3.1
Tabletop structure	30 × 30 × 50	4.4

**Table 2:** Characteristics of the spectrometers: grating, effective and used wavelength range, and spectral resolution.

No.	Grating (gr/mm)	Effective Range (nm)	Used Range (nm)	Resolution (nm)
1	3600	178–259	180–250	0.06
2	3600	248–318	250–311	0.07
3	2400	307–423	311–418	0.11
4	1800	407–561	418–555	0.11
5	1800	546–670	555–668	0.12
6	1200	646–875	668–675	0.16

**Table 3:** List of representative samples used for testing of the LIBS instrument.

No.	Sample	Type	Other	Comments
1	Wafer substrate	Clean	-	Instrument check
2	21ST	Printed spots on wafer	500 pg per element	Signal optimization, LOD
3	21ST	Printed spots on wafer	100 pg per element	LOD
4	21ST	Printed spots on wafer	10 pg per element	LOD
5	Sebaceous fingerprint	Fingerprint on wafer	Unknown mass	Detection feasibility
6	Soil NIST 2710	Swabbed on wafer	Unknown mass	Detection feasibility
7	GSR	Swabbed on wafer	From gun	Detection feasibility
8	White car varnish	Bulk	Coating 100 µm	Depth profiling
9	White car varnish	Bulk	Coating 35 µm	Depth profiling
10	Black ABS	Bulk	FR coating	Depth profiling

**Table 4:** Analytical lines of elements detected on 21ST sample: wavelength, excitation energy E_k_, and the average SNR measured for the three tested element masses. The last column reports RSD of the analytical peaks for element mass of 100 pg. The acquisition delay was 2500 ns.

Element	Line (nm)	Ek (eV)	Notes	SNR 500 pg	SNR 100 pg	SNR 10 pg	RSD 100 pg
Al I	309.27	4.022	Impurity	39	40	3.7	0.21
B I	249.77	4.964	Sample	22	32	39	0.061
Be II	313.04	3.959	Sample	228	293	93	0.080
C I	247.86	7.684	Impurity	26	40	22	0.068
Ca I	422.67	2.932	Sample	84	149	62	0.12
Cd I	228.80	5.417	Sample	14	13	3.5	0.082
Co I	345.35	4.021	Sample	28	26	5.8	0.11
Cr I	425.44	2.913	Sample	70	61	14	0.084
Cu I	324.75	3.817	Sample	55	52	42	0.085
Fe I	404.58	4.548	Sample	17	17	3.5	0.079
H I	656.28	12.088	Air	17	35	22	0.12
Li I	670.78	1.848	Sample	292	225	170	0.062
K I	769.90	1.610	Impurity	11	33	19	0.083
Mg I	285.21	4.345	Sample	45	46	57	0.13
Mn I	403.08	3.075	Sample	38	43	7.1	0.076
Mo I	550.65	3.586	Sample	47	22	3.9	0.14
N I	746.83	11.995	Air	41	46	67	0.15
Na I	589.59	2.102	Impurity	41	37	65	0.075
Ni I	341.48	3.655	Sample	13	10	5.9	0.079
O I	777.19	10.741	Air	239	198	336	0.20
Pb I	405.78	4.375	Sample	34	17	-	0.075
Sb I	259.80	5.826	Sample	7.3	7.9	-	0.26
Si I	288.16	5.082	Wafer	328	300	1243	0.12
Sr II	407.77	3.040	Sample	365	338	61	0.17
Ti I	499.11	3.319	Sample	26	22	3.9	0.090
V I	437.92	3.131	Sample	87	24	5.1	0.10
Zn I	213.86	5.796	Sample	5.9	5.4	-	0.14

I = atomic state. II = first ionization.

## Data Availability

The data of the measurements that support the findings of this study are available from the corresponding author upon reasonable request.
